# A rare maxillary lesion: a case report of developing complex odontoma in the left maxilla of a 5-year-old child

**DOI:** 10.3389/fdmed.2026.1760785

**Published:** 2026-04-13

**Authors:** Junji Xu, Lanqiu Lv, Yiduo Shao, Shanshan Guo

**Affiliations:** 1 The Affiliated Women and Children's Hospital of Ningbo University, Ningbo, China; 2 Ningbo Hospital of Integrated Traditional Chinese and Western Medicine, Otolaryngology Head and Neck Surgery, Ningbo, China

**Keywords:** cone-beam computed tomography, developing complex odontoma, left maxillary mass, maxillary tumor, odontoma

## Abstract

**Objective:**

This study aims to report the clinical diagnosis and management of a 5-year-old child with developing complex odontoma in the left maxilla, and to review the literature regarding its clinical features, imaging findings, and pathological characteristics, so as to provide clinical insights for early diagnosis and individualized treatment of pediatric maxillary developing complex odontoma.

**Materials and methods:**

A 5-year-old male presenting with a “left maxillary mass” was evaluated. Clinical examination assessed facial morphology and dental development. Panoramic radiography and cone-beam computed tomography (CBCT) were used to determine the lesion's location and imaging characteristics. The surgically resected specimen was sent to the Ningbo Clinical Pathological Diagnosis Center for gross and histopathological examination. Postoperative follow-up was conducted for 24 months to monitor wound healing, dental development, and potential recurrence. We implemented a proactive monitoring approach, which included regular follow-up visits every three months during the first year. During these visits, we assessed the growth and development of the surrounding teeth and evaluated the edentulous area for any signs of potential complications.

**Results:**

Clinical examination revealed a left maxillary mass without significant facial asymmetry, and the child was in the stage of dental development. Imaging confirmed an expansive lesion in the left maxilla. Pathological examination identified a 4.5 × 4 × 0.5 cm grayish-white to reddish, firm tissue, leading to a final diagnosis of developing complex odontoma. The lesion was completely removed via curettage. At the 24-month follow-up, no recurrence was observed, the surgical site had healed well, dental development was normal, and no complications such as secondary caries were noted.

**Conclusion:**

Pediatric maxillary developing complex odontoma often presents with subtle symptoms and atypical imaging features, leading to delayed diagnosis. Multimodal imaging combined with pathological examination enables accurate diagnosis. Complete excision via curettage, accompanied by long-term follow-up, effectively restores maxillary structure and function, supports normal dental development, and reduces recurrence risk, making it a viable treatment strategy for pediatric maxillary developing complex odontoma.

## Introduction

Developing complex odontoma is a benign odontogenic tumor with bidirectional differentiation, histologically characterized by the presence of odontogenic epithelial strands, embryonic mesenchyme, and dental hard tissues. Epidemiological studies indicate a clear predilection for age and site: 92.6% of cases occur in patients under 20 years old, while only 15.7% occur in the maxilla (1). Notably, fewer than 30 cases of pediatric maxillary developing complex odontoma have been reported globally, accounting for only 2.8% of all developing complex odontoma cases (2). Lesions in this anatomical site are often diagnosed late due to their insidious onset and nonspecific radiological appearance (3). This report presents a case of developing complex odontoma in the left maxilla of a 5-year-old male, integrating multimodal imaging and pathological findings to aid in early recognition and treatment planning for complex odontogenic tumors in children.

## Case presentation

A 5-year-old boy presented with the chief complaint of a “left maxillary mass.” He had no significant past medical history. Clinical examination revealed a left maxillary mass without noticeable facial asymmetry. Intraoral examination showed the child was in the stage of mixed dentition. A provisional diagnosis of a maxillary tumor was made.

**Figure 1:**
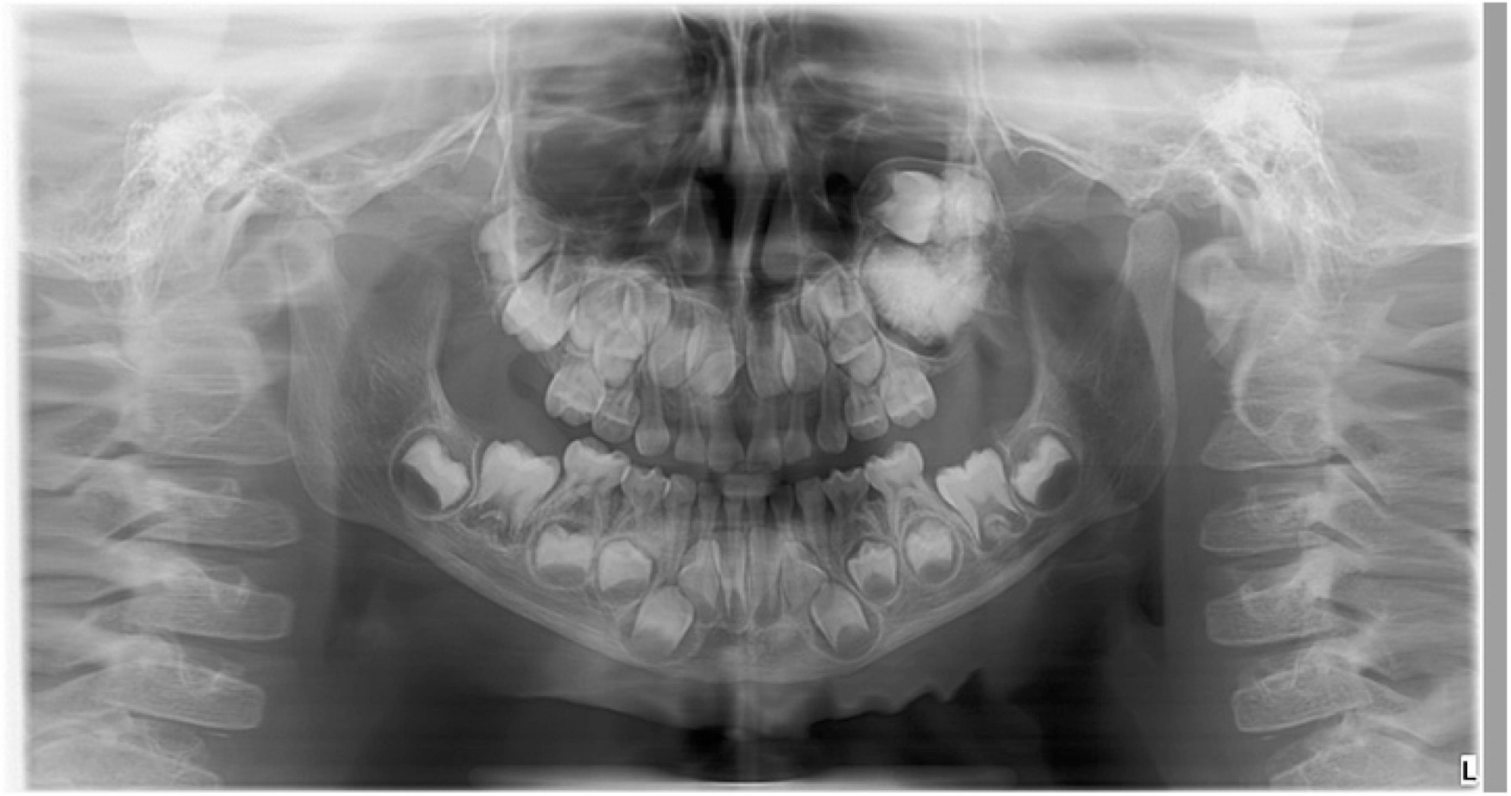
Preoperative panoramic radiograph.

**Figure 2:**
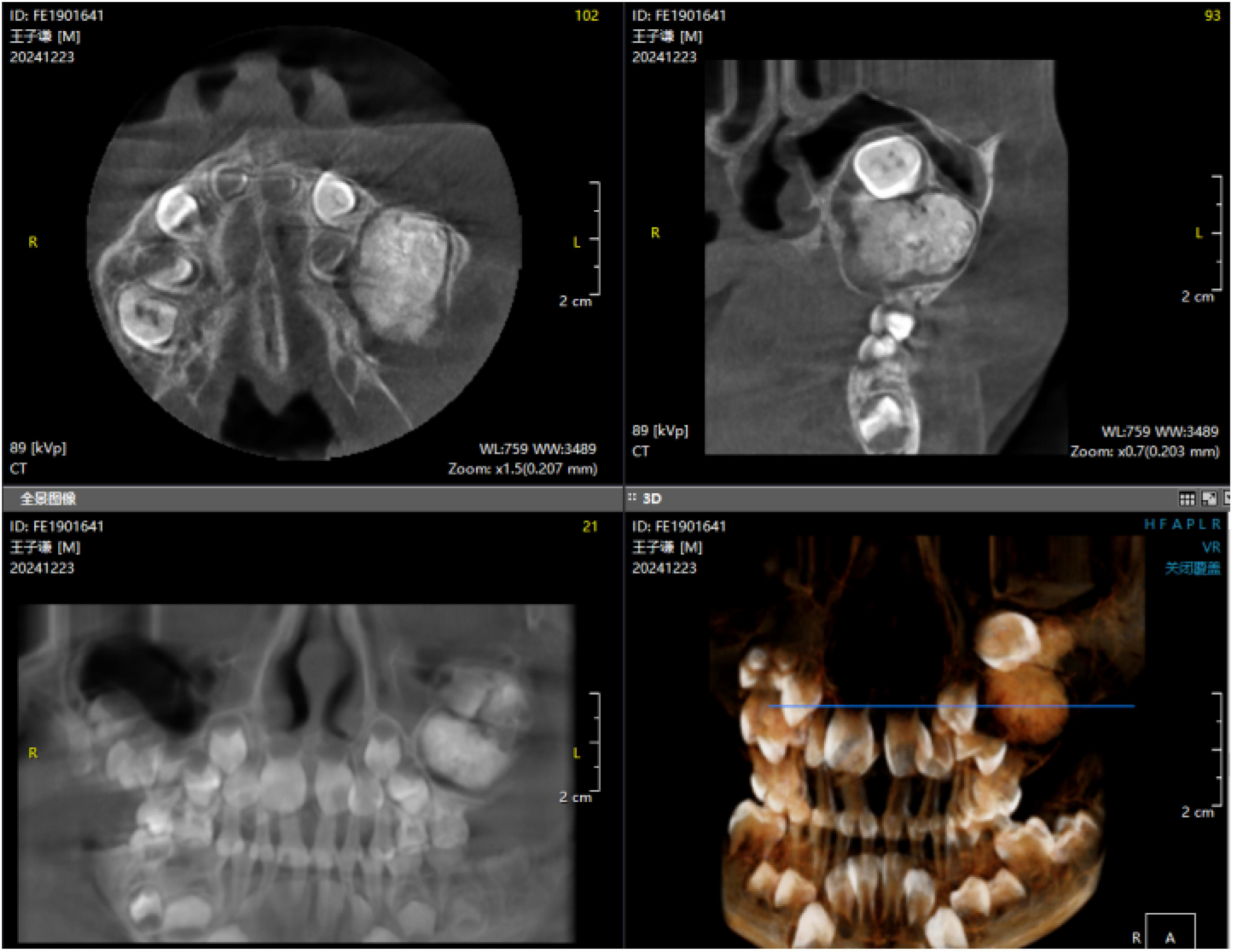
Preoperative CBCT image (1).

**Figure 3:**
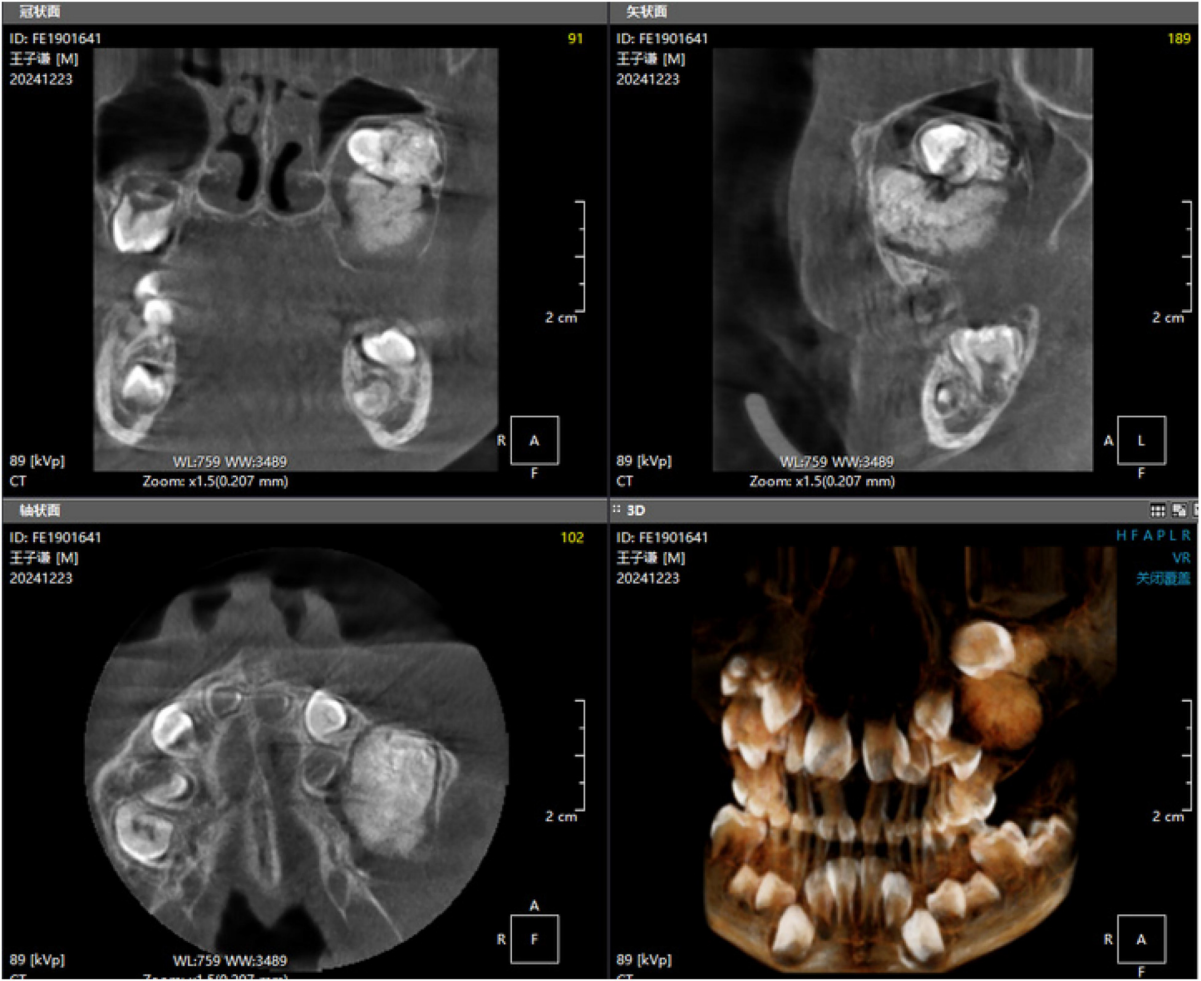
Preoperative CBCT image (2).

Panoramic radiography and CBCT confirmed the lesion located in the left maxilla (Figures 1–3).

**Clinical Diagnosis:** Maxillary tumor.

### Treatment objectives

To eradicate the developing complex odontoma in the left maxilla, restore normal maxillary structure and function, ensure normal dental development, and address potential complications such as secondary caries.

### Treatment plan

Surgical excision of the left maxillary developing complex odontoma was planned. Follow-up examinations were scheduled to monitor surgical site healing and dental development. Secondary caries would be assessed and managed as needed after tumor removal and stabilization of dental development. Evaluate whether to select and implement interventions for the child in terms of orthodontics, denture planning, growth monitoring programs, and speech therapy in the future.

### Treatment process

**Figure 4:**
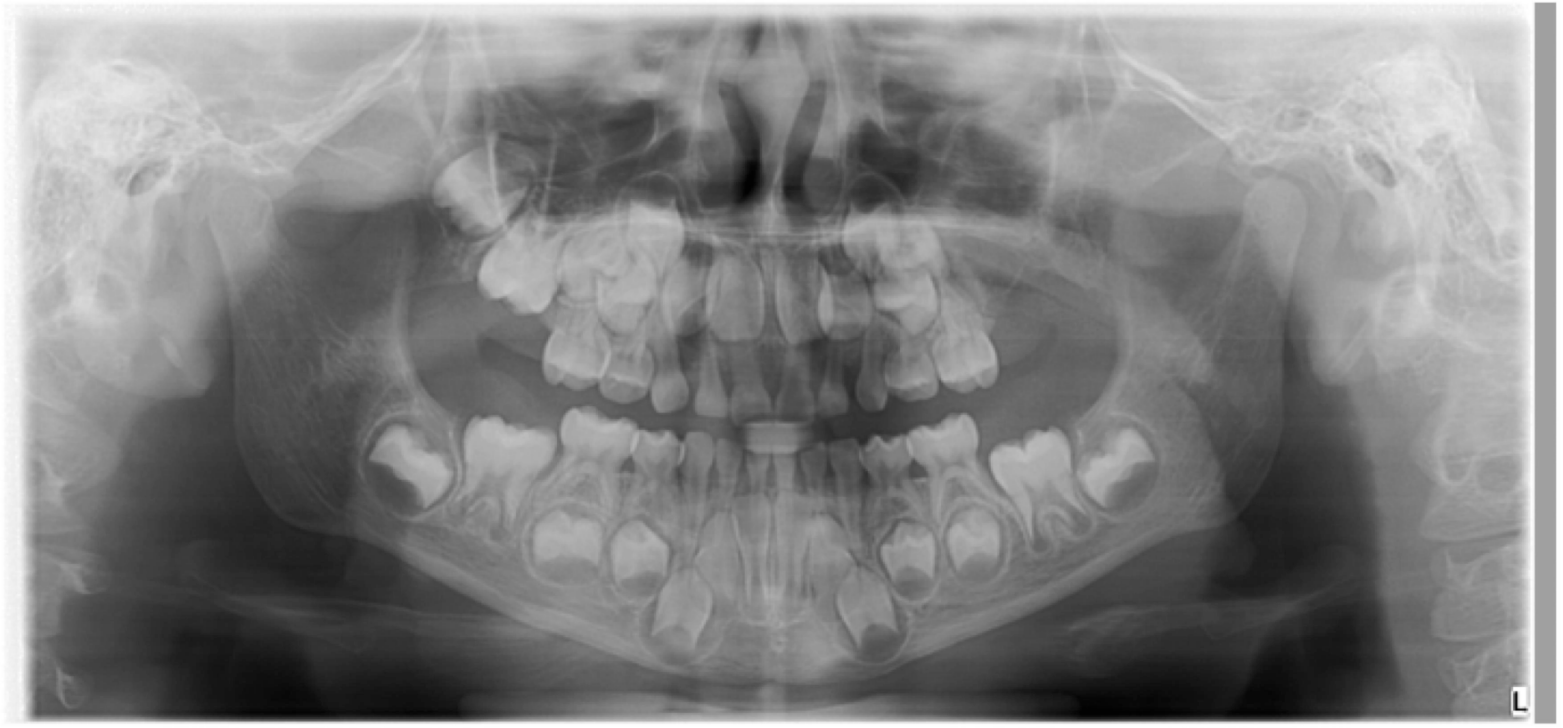
Postoperative panoramic radiograph.

**Figure 5:**
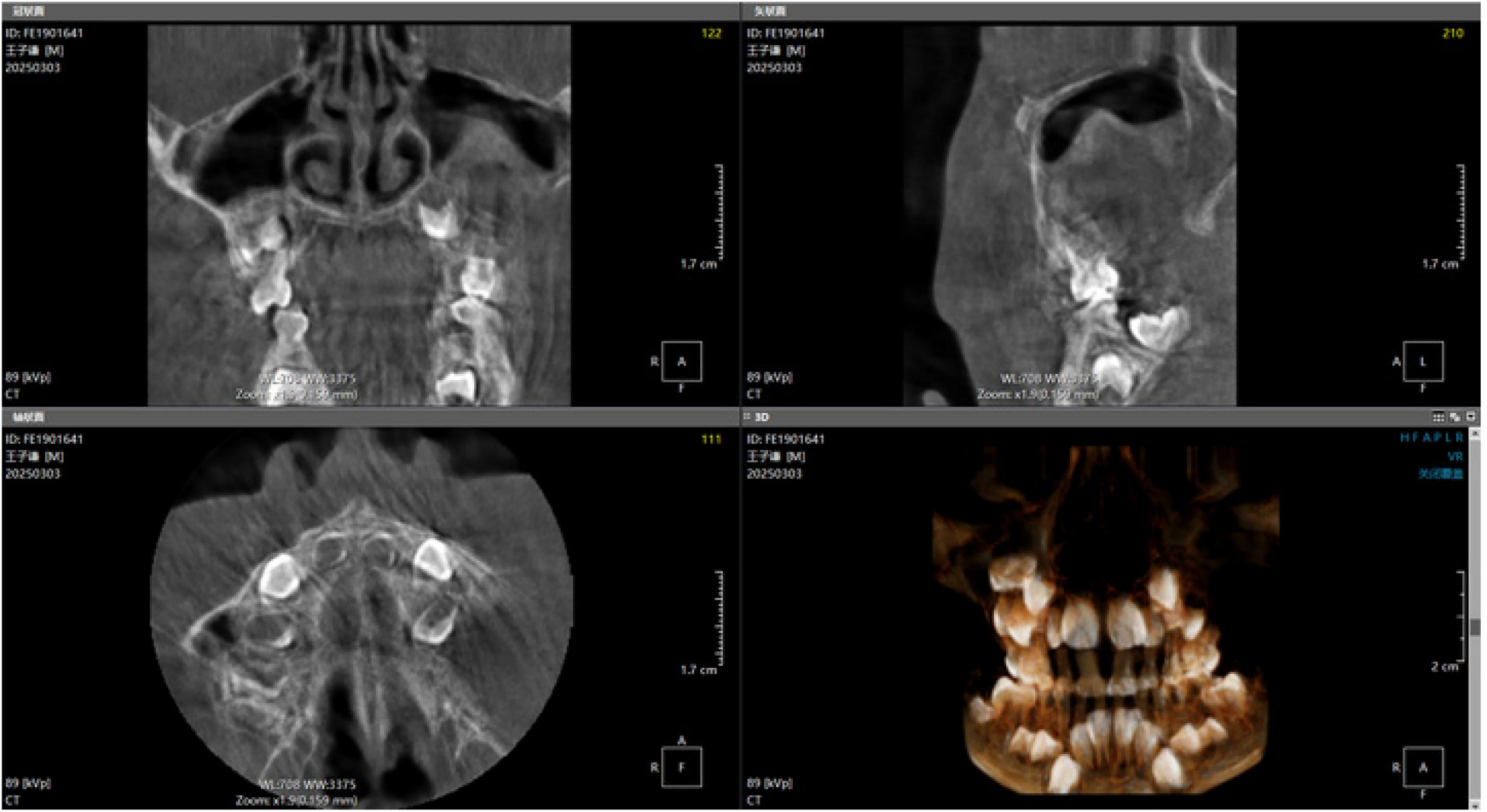
Postoperative CBCT image (1).

**Figure 6:**
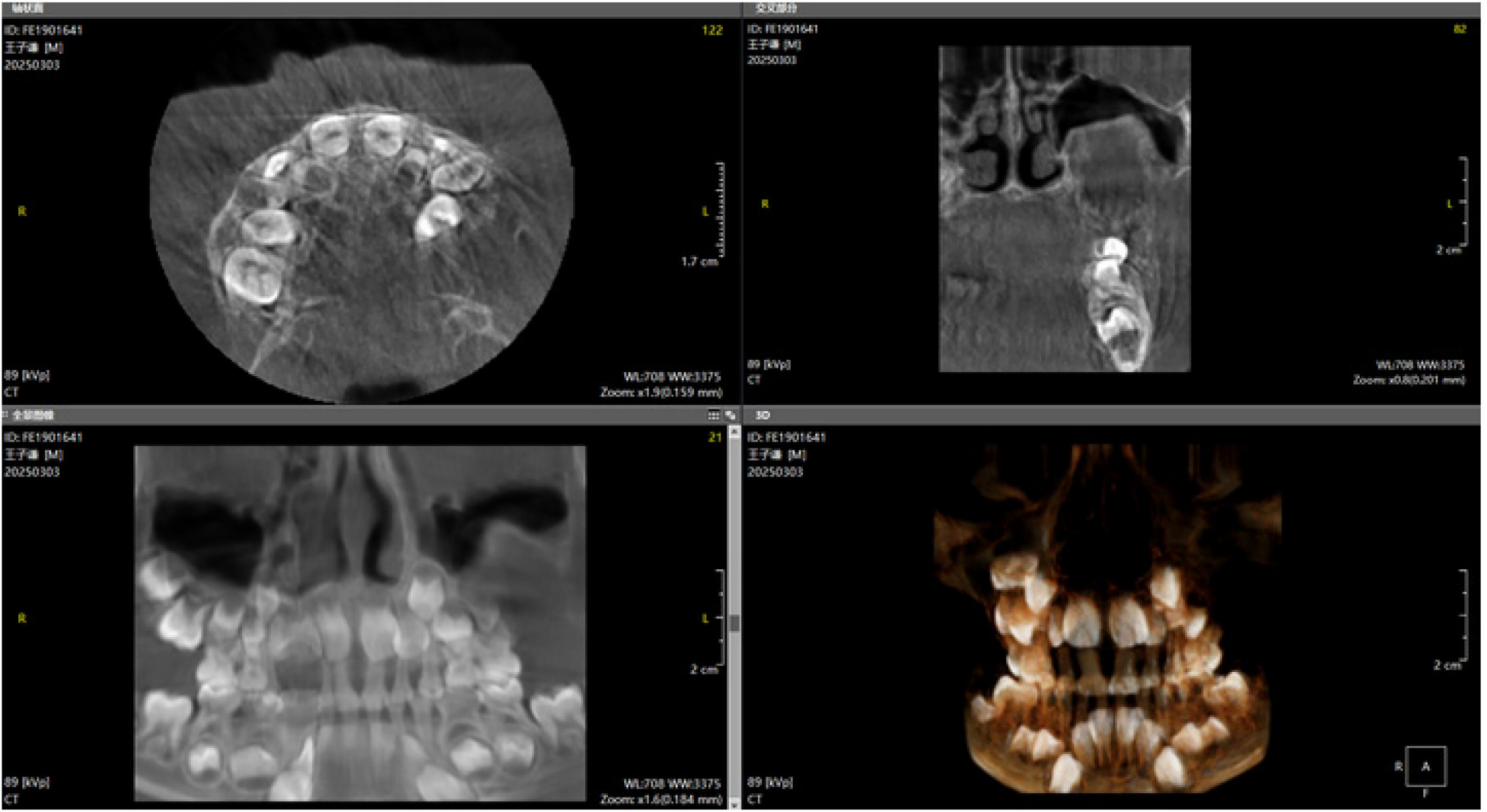
Postoperative CBCT image (2).

The left maxillary developing complex odontoma was surgically excised (Figures 4–6). Postoperatively, the surgical site was closely monitored to ensure proper healing. Regular follow-up assessments evaluated dental development. After adequate healing and stabilization, potential secondary caries were assessed and managed as required. Throughout treatment, measures were taken to restore and maintain normal maxillary structure and function. At the 6-month postoperative re-examination, the child's facial contour was basically symmetrical, and the occlusal relationship was acceptable. Considering that the child was in the primary dentition stage, orthodontic intervention will be gradually performed in the later stage according to the child's jaw growth and occlusal changes. Considering that the child was in the primary dentition stage with continuous jaw growth, the necessity of denture restoration will be evaluated after the eruption of the child's permanent teeth to ensure masticatory function and facial appearance. The child's speech and pronunciation were all normal. Dental development parameters: The eruption of the left maxillary primary teeth was normal at re-examination.

### Pathological diagnosis

(Left maxillary tumor) developing complex odontoma. Developing complex odontomas are characterized by a disorganized arrangement of enamel and dentin-like tissues with varying degrees of mineralization. They typically exhibit a mixture of dental hard tissues, including enamel prisms and tubular structures, often surrounded by a fibrous capsule, reflecting their developmental stage.

### Differential diagnosis

1. Composite Odontoma: Unlike complex odontomas, composite odontomas contain well-formed dental tissues, such as enamel and dentin, in a more organized manner, resembling actual teeth. They typically present as distinct, tooth-like structures.
2. Ameloblastoma: This benign, aggressive odontogenic tumor may exhibit similar radiographic features, such as multilocular radiolucencies. However, ameloblastomas are characterized by the presence of neoplastic epithelial cells and lack the mineralized dental tissue composition seen in odontomas.

**Figure 7:**
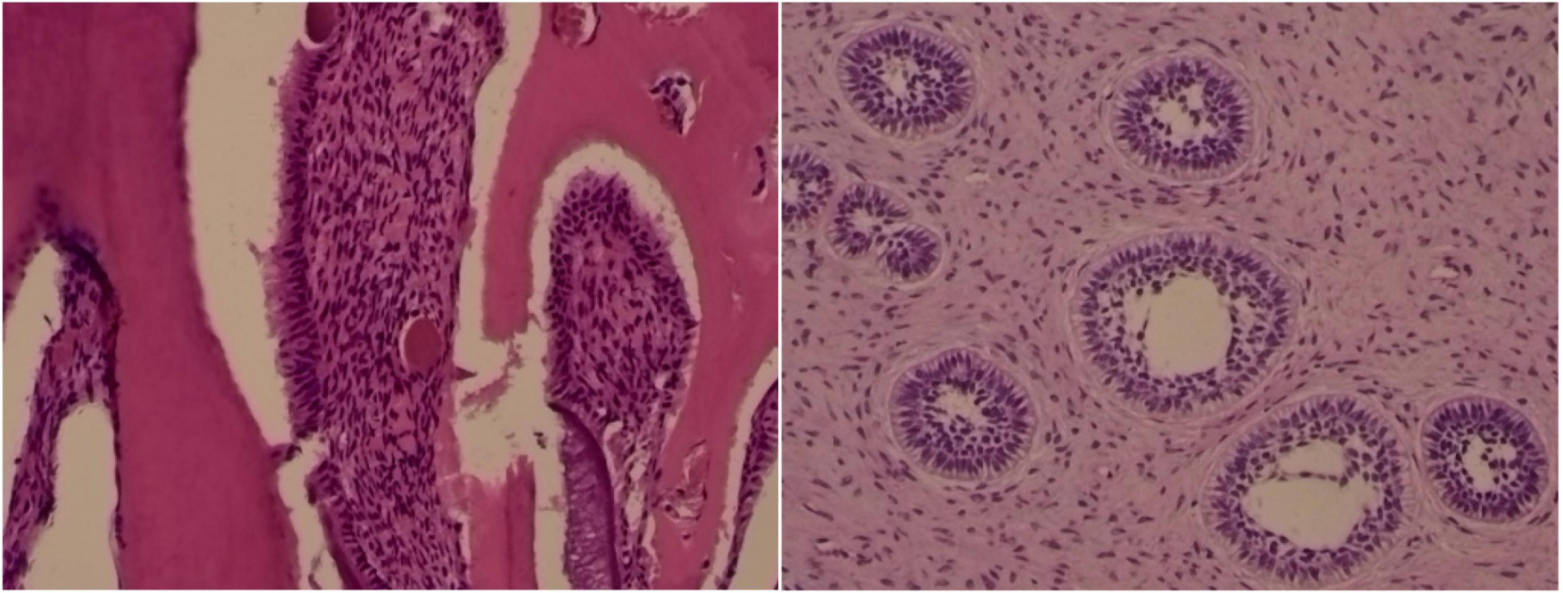
Hematoxylin and eosin (H&E) staining of the left maxillary mass.

The specimen was submitted to the Ningbo Clinical Pathological Diagnosis Center. Gross description: (Left maxillary tumor) multiple grayish-white to reddish tissue fragments, measuring 4.5 × 4 × 0.5 cm in aggregate, firm in consistency, submitted in entirety (Figure 7).

## Discussion

Developing complex odontoma is an odontogenic tumor originating from the dental germ or mesenchymal components of the dental papilla (4). Early detection and treatment are crucial in pediatric patients to prevent impacts on maxillary development and dentition.

Surgical excision is the primary treatment for early-stage developing complex odontoma. Complete removal of tumor tissue is essential to minimize recurrence risk (5). In this case, curettage was chosen for complete excision, offering advantages over more extensive resection, such as reduced trauma and less impact on jaw and dental development (6). Stability of the surgical site and normal dental development are key indicators of treatment success. Postoperative follow-up is necessary to monitor for recurrence and assess maxillary and dental development. Self-growth compensation ability of the jaw: Children aged 5 have great potential for jaw growth and are in a period of rapid growth and development. The remaining jaw tissue after surgery can achieve self-repair and growth through cell proliferation and bone deposition, gradually filling the defect area and improving the problem of insufficient jaw volume. Adaptive adjustment of surrounding soft tissues and muscles: The masticatory muscles and facial expression muscles attached around the maxilla can adjust through their own tension to adapt to the changes in jaw volume after surgery, avoiding severe abnormalities in facial contour caused by volume reduction. At the same time, the functional contraction of muscles can stimulate jaw growth, guide the normal development of the jaw, assist in improving facial symmetry, and reduce adverse effects on the temporomandibular joint.

Compared to other odontogenic tumors, developing complex odontoma generally has a favorable prognosis, though long-term follow-up is recommended to ensure no residual tumor cells persist. Additionally, managing potential complications like secondary caries is an integral part of comprehensive care. Treatment should be tailored to tumor characteristics, including location, size, histology, and the patient's overall health and developmental stage. In this pediatric case, treatment balanced effective tumor removal with preservation of normal maxillary and dental development. Further research is needed to explore long-term outcomes and optimize treatment strategies for pediatric developing complex odontoma.

## Data Availability

The data that support the findings of this study are available from the corresponding author upon reasonable request.

## References

[1] Surej Kumar LK Manuel S Khalam SA Venugopal K Sivakumar TT Issac J. Ameloblastic fibro-odontoma. Int J Surg Case Rep. (2014) 5(12):1142–4. 10.1016/j.ijscr.2014.11.025 25437658 PMC4276268

[2] Hendra FN Van Cann EM Helder MN Ruslin MH De Visscher JG Forouzanfar T Global incidence and profile of ameloblastoma: a systematic review and meta-analysis. Oral Dis. (2020) 26:12–21. 10.1111/odi.13031 30614154

[3] Boos-Lima FBDJ Sutikno I Bell RB Peacock ZS. Odontogenic and non-odontogenic tumors of the jaws. In: Andersson L Krishnan DG Peacock ZS, editors. Oral and Maxillofacial Surgery. Hoboken, NJ: John Wiley & Sons Ltd (2025). 10.1002/9781119842958.ch5.3

[4] Akinosi JO Olufemi Williams A. Ameloblastoma in Ibadan, Nigeria. Oral Surg Oral Med Oral Pathol. (1969) 27(2):257–65. 10.1016/0030-4220(69)90181-9 ISSN 0030-4220. 5249523

[5] Goudarzi S Cinquini C Izzetti R Nisi M Priami M Brevi BC Oral rehabilitation following surgical treatment of mandibular ameloblastoma: case report and comprehensive literature review. Oral. (2025) 5(3):57. 10.3390/oral5030057

[6] Xu C Hu Y Sun Y Shao Q Song Y He J. Curettage combined with decompression for the treatment of ameloblastoma in children: report of two cases. BMC Oral Health. (2024) 24(1):378. 10.1186/s12903-024-04126-8 38519948 PMC10958900

